# The effect of transversus abdominis plane block on the chronic pain after colorectal surgery: a retrospective cohort study

**DOI:** 10.1186/s12871-020-01032-8

**Published:** 2020-05-18

**Authors:** Zi-Ye Pan, Zhong-Hua Hu, Fan Zhang, Wen-Xiu Xie, Yong-Zhong Tang, Qin Liao

**Affiliations:** Department of Anesthesiology, The Third Xiangya Hospital of Central South University, Changsha, 410013 Hunan China

**Keywords:** Chronic postsurgical pain, Colorectal surgery, Transversus abdominal plane block, Analgesia

## Abstract

**Background:**

Chronic postsurgical pain (CPSP) is common and would reduce the quality of life of patients. Transversus abdominal plane (TAP) block has been widely used in lower abdominal surgery and many researches demonstrated that it could improve acute postsurgical pain. We aim to determine whether TAP block could improve chronic postoperative pain at 3 months and 6 months after colorectal surgery.

**Methods:**

A total of 307 patients received selective colorectal surgery under general anesthesia between January, 2015 and January, 2019 in a single university hospital were included: 128 patients received TAP block combined with patient-controlled intravenous analgesia (PCIA) for postsurgical analgesia (group TP) and 179 only administrated with PCIA (group P). Main outcome was the NRS score of pain at 3 months after colorectal surgery. The data was analyzed by two-way repeated measures anova and the chi-square test.

**Results:**

The NRS score at rest and during movement was decreased significantly at 24 h after surgery (rest NRS 1.07 ± 1.34 vs 1.65 ± 1.67, movement NRS 3.00 ± 1.45 vs 3.65 ± 1.89; all *P* = 0.003) in group TP than those of group P. There was no significant difference of NRS score at 48 h after surgery (*P* > 0.05). At 3 months after surgery, the NRS score during movement was also lower in group TP than that in group P (0.59 ± 1.23 vs 0.92 ± 1.65, *P* = 0.045). There was no significant difference of NRS score at 6 months after surgery (*P* > 0.05).

The prevalence of CPSP was 19.5% (25/128) in group TP and 20.7% (37/179) in group P at 3 months after surgery. 13.2% (17/128) of patients suffered from CPSP in group TP and 13.9% (25/179) in group P at 6 months after surgery. Both at 3 months and 6 months after surgery, there was no statistical difference of the prevalence of CPSP between the two groups (all *P* > 0.05) .

**Conclusions:**

TAP block reduced NRS during movement at 3 months after surgery but did not reduce the incidence of CPSP at 3 months and 6 months after selective colorectal surgery.

## Background

Chronic postsurgical pain (CPSP) is defined by the International Association for the Study of Pain (IASP) as pain that develops after a surgical procedure and persists for at least 3 months after surgery, where all other causes of pain (e.g. infection, recurring malignancy) or pre-existing pain problems are excluded [1, 2]. Few studies reported the prevalence of CPSP after colorectal surgery [3, 4]. A prospective study showed that 19% of patients underwent colorectal surgery reported CPSP at 6 months, 63% of which taking medication for their pain, 53% of which were using opioids to manage their pain [3]. In another retrospective study, 17% of patients reported CPSP after laparoscopic colorectal surgery, 37% of which suffering from severe pain (pain score ≥ 7) [4]. CPSP has a negative impact on the quality of life, such as mood, sleep, walking ability, and normal work, so it is important to find out methods to reduce CPSP.

It was found that acute postoperative pain was an important risk factor of CPSP, [4–6] and many researches demonstrated that CPSP could be partly improved by optimizing perioperative pain management, including intravenous analgesia, epidural analgesia, paravertebral blocks and regional analgesia [7–9]. Transversus abdominis plane (TAP) block is an effective regional analgesia technique to reduce perioperative pain intensity, [10] especially combined with ultrasound-guided technique. TAP block is the injection of local anesthetics to neurofascial plane between internal oblique and transversus abdominis muscles aiming at blocking the neural afferents from the anterolateral abdominal wall (T7-L1) [11]. TAP block has been widely used in many abdominal operation, with a satisfactory duration of analgesic action for up to 24 h after surgery [10, 12–14]. Many studies confirmed that TAP block can improve acute postoperative pain, [15, 16] but whether TAP block can reduce chronic pain after colorectal surgery has been reported scarcely.

The main aim of our study was to evaluate whether TAP block could improve CPSP after colorectal surgery. We compared the NRS score of different time point for patients who had TAP block and PCIA with those patients without TAP block.

## Methods

Our research was approved by the Ethics Committee of Third Xiangya Hospital, Central South University (No:2019-S473). We reviewed the medical records of patients undertaking colorectal surgery at the third Xiangya hospital of Central South University from January, 2015 to January 2019. Eligibility criteria included patients who were over 18 years old, American Society of Anesthesiologists (ASA) status I-III, receiving selective colorectal surgery under general anesthesia and PCIA for postsurgical analgesia. Exclusion criteria were patients unable to communicate, wound infection after surgery, receiving a second operation.

### Study protocol

On entering the surgery room, patients’ vital signs were monitored by electrocardiogram, invasive arterial blood pressure, pulse oximetry and bispectral index. General anesthesia was induced with fentanyl (4–6 μg/kg) or sufentanil (0.4–0.6 μg/kg), propofol (1.5–2 mg/kg), midazolam (2–3 mg), rocuronium (1 mg/kg) or cisatracurium besylate (0.15–0.2 mg/kg). Anesthesia was maintained both with intravenous infusion propofol 4 to 7 mg/kg/h, remifentanil 6 to 10 μg/kg/h, and inhalation of 1 to 2% sevoflurane. Fentanyl or sufentanil was used when signs of inadequate analgesia were evident intraoperatively such as increased heart rate or blood pressure. 0.05 mg/kg of cisatracurium was administered at 30–40 min intervals. All patients received 0.25 mg palonosetron hydrochloride intravenously before suture.

For those patients who received TAP block, bilateral TAP block was performed by ultrasound (SonoSite EdgeII, American) before anesthesia induction. 40 ml of 0.5% ropivacaine (AstraZneca AB, Sweden, 20 ml each side) was injected into the transversus abdominis plane under real-time ultrasound guidance.

Postoperatively, electronic analgesic pump with wireless analgesic system was used for 48 h of PCIA (Renxian Medtech, Jiangsu). The PCIA pump was filled with sufentanil 150 μg, azasetron 10 mg, diluted to 150 ml with 0.9% normal saline. It was programmed to give 1.5–2 ml/h background infusion with a 1.5–2 μg bolus of sufentanil solution, with a 5 min lockout time. Patients were routinely informed that they could control the pain by pressing a self-controlled button to only slight pain. Pain was assessed twice a day at rest and during movement with an 11-point Numeric Rating Scale (NRS) (0 = no pain; 10 = pain ‘as bad as you can imagine’) by the same nurse of acute pain service group within 48 h after operation.

After discharged from hospital, postoperative follow-up team contacted patients via telephone at 3 months and 6 months after operation to complete pain evaluation (NRS score and painful place).

### The data extraction

Patients’ data including patients’ ASA status, sex, age at the time of operation, body mass index was extracted from medical records. Patients’ information about surgical and anesthetic management such as anesthesia duration, surgery duration, perioperative opioid dose and pain scores was extracted from Anesthesia records. All patients were allocated to two groups: group TP were those patients who received TAP block and PCIA; and group P were those patients who only received PCIA.

### Statistical analysis

In order to facilitate comparison, fentanyl 0.1 mg was equivalent to sufentanil 0.01 mg. Considering that remifentanil is an ultra-short-acting opioid, we analyzed it separately. Opioid usage was analyzed with the unpaired t-test between two groups. Two-way repeated measures anova was used for NRS scores of 24 h, 48 h, 3 months and 6 months after surgery. The chi-square test was used for comparisons of categorical data. Quantitative data were expressed as mean ± SD and categorical variables as percentages. Data were analyzed with the SPSS software version 22.0 (SPSS Inc., Chicago, IL). Significance was determined at P<0.05.

### Patient and public involvement

Patients and the public were not involved in planning, design, or interpretation of the study. The study involved examination of existing claims data and no participants were recruited for this analysis. This research was done without patient involvement. Patients were not invited to comment on the study design and were not consulted to develop patient relevant outcomes or interpret the results. Patients were not invited to contribute to the writing or editing of this document for readability or accuracy.

## Results

**Fig. 1:**
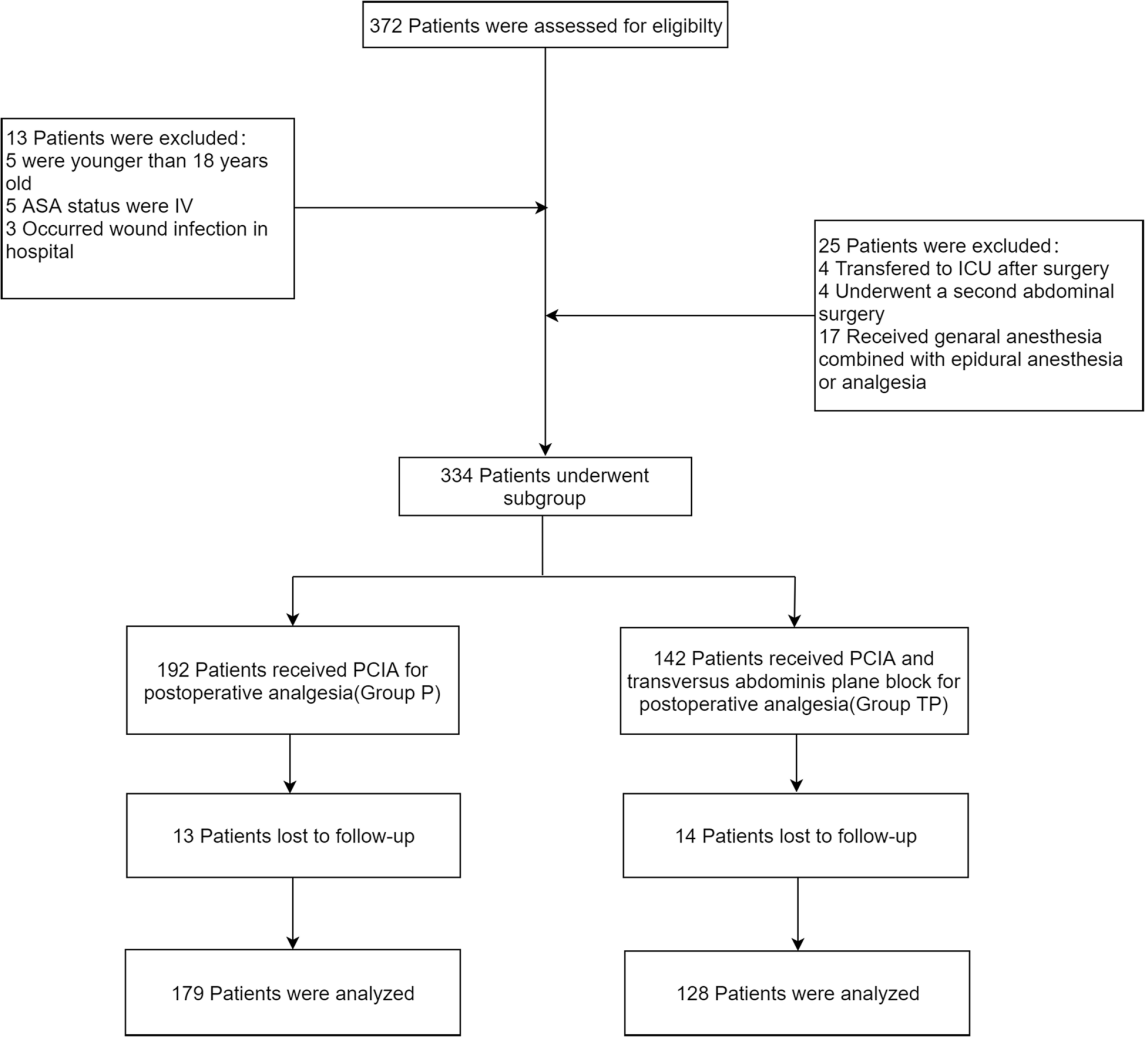
Flow chart. Legend: Flow chart showing patients enrollment, grouping and analysis

Three hundred-seventy-two patients were included in this study between January 2015 and January 2019. Thirty eight patients were excluded (5 patients were younger than 18 years, 5 patients’ ASA status was IV, 3 patients occurred wound infection in hospital, 4 patients were transferred to ICU after surgery, 4 patients underwent a second abdominal operation, 17 patients received general anesthesia combined with epidural anesthesia or analgesia). Three hundred-thirty-four patients meeting the selection criteria were reviewed (group *P* = 192; group TP =142). There were 13 patients lost to follow-up in group P and 14 patients in group TP. Finally, 307 patients were enrolled for analysis. The participant flow chart of this study was presented in Fig. 1.

Patients’ demographics, ASA status, duration of surgery and anesthesia, and operative method were similar between the two groups (Table 1).

**Table 1:** Patients’ demographics and clinical characteristics

	Group P (*n* = 179)	Group TP (*n* = 128)	Total (*n* = 307)	*P*
Gender				
Male	79(44.1%)	74(57.8%)	153(49.8%)	0.734
Female	100(55.9%)	54(42.2%)	154(50.2%)	
Age (years)	57.8 ± 11.8	59.6 ± 12.2	58.6 ± 12.0	0.190
BMI (kg/m^2^)	22.7 ± 3.2	22.3 ± 2.8	22.5 ± 3.0	0.284
ASA status				
I	1(0.6%)	1(0.8%)	2(0.7%)	0.405
II	102(57.0%)	73(57.0%)	175(57.0%)	
III	76(42.4%)	54(42.2%)	130(42.3%)	
Surgery duration (hours)	3.8 ± 1.4	3.9 ± 1.0	3.8 ± 1.3	0.671
Anesthesia duration (hours)	5.5 ± 1.4	5.6 ± 1.0	5.5 ± 1.3	0.593
Operative Method				
Open	36(20.1%)	16(12.5%)	52(16.9%)	0.080
Laparoscope	143(79.9%)	112(87.5%)	255(83.1%)	

Measurement data is presented as Mean ± SD or number (%). Group P=PCIA only, Group TP = PCIA and TAP block

Compared with group P, there was a significant reduction in intraoperative sufentanil usage in group TP (51.3 ± 12.6 vs 64.8 ± 18.8, *P* = 0.000), while there was no significant difference in remifentanil usage between two groups *(P* > 0.05). But the postoperative requirement for sufentanil showed no significant difference at 24 h and 48 h between group TP and group P (*P* = 0.255, *P* = 0.300, respectively) (Table 2).

**Table 2:** Opioid usage during perioperative period

Opioid usage	Group P (*n* = 179)	Group TP (*n* = 128)	*t*	*P*
Intraoperative				
Sufentanil (ug)	64.8 ± 18.8	51.3 ± 12.6	−7.071	0.000*
Remifentanil (mg)	1.4 ± 0.6	1.3 ± 0.5	−1.831	0.068
Postoperative				
Sufentanil usage at 24 h (ug)	45.1 ± 30.2	49.3 ± 34.0	1.141	0.255
Sufentanil usage at 48 h (ug)	84.7 ± 49.1	90.6 ± 50.3	1.038	0.300

Data are presented as Mean ± SD, compared with Group P, *P<0.05. Group P=PCIA only, Group TP = PCIA and TAP block

**Fig. 2:**
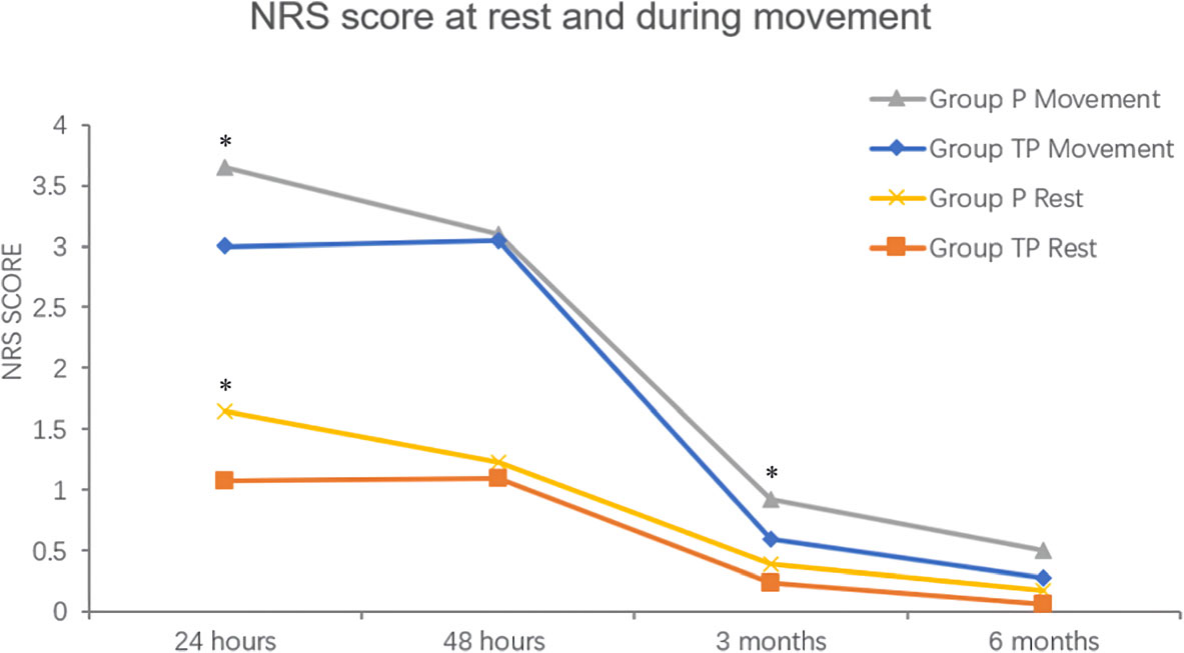
NRS score at rest and during movement. Legend: The NRS score of patients at rest and during movement at different time points. Data is presented as mean ± SD. Compared with group P, the NRS score (at rest and during movement) was decreased significantly at 24 h in group TP (*P*<0.05), but there was no significant difference at 48 h between two groups (*P* > 0.05). In group TP, the NRS score during movement was significantly lower than that in group P at 3 months after surgery (*P*<0.05). There were no significant difference of NRS score at 6 months after surgery between two groups (*P* > 0.05). NRS = numerical rating scale. Group P=PCIA only, Group TP = PCIA and TAP block. * *P*<0.05

The NRS score at rest and during movement was decreased significantly at 24 h after surgery (rest NRS 1.07 ± 1.34 vs 1.65 ± 1.67, movement NRS 3.00 ± 1.45 vs 3.65 ± 1.89; all *p* = 0.003) in group TP than those of group P. but there was no significant difference of NRS score at rest and during movement at 48 h (*p* > 0.05). At 3 months after surgery, the NRS score during movement was also lower in group TP than that in group P (0.59 ± 1.23 vs 0.92 ± 1.65, *p* = 0.045). There was no significant difference of NRS score at 6 months after surgery (all *P* > 0.05) (Fig. 2).

In time comparison, both in group TP and group P, the effect of time on pain was statistically significant (all *P* < 0.001) and the degree of pain decreased with time (Fig. 2).

**Fig. 3:**
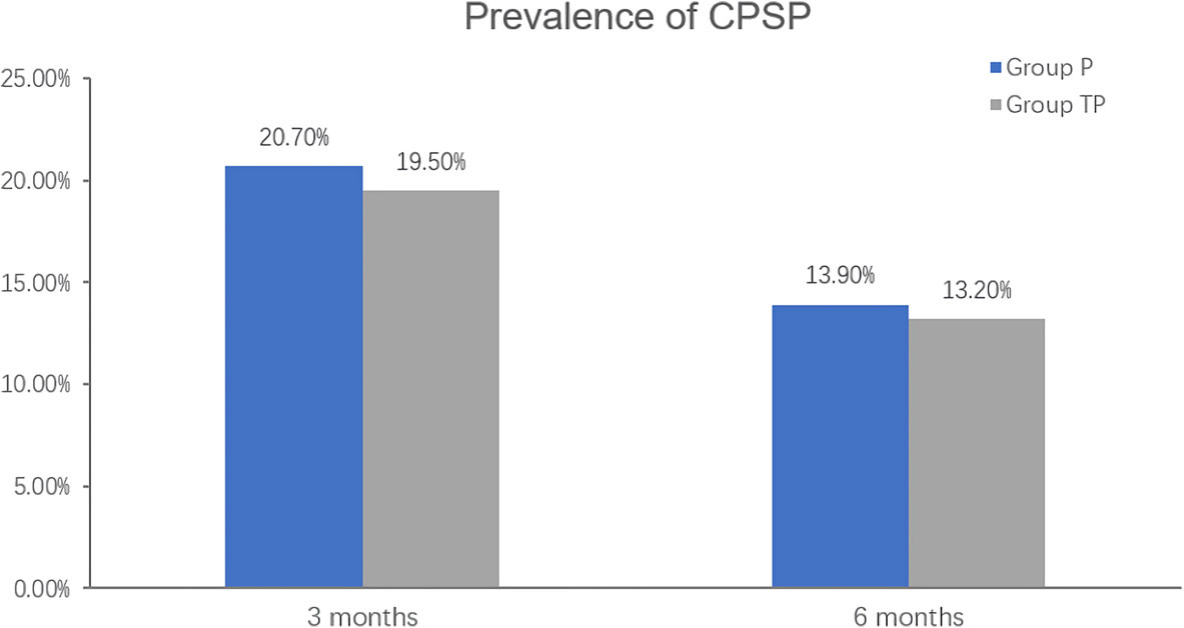
Prevalence of CPSP. Legend: The prevalence of CPSP at 3 months and 6 months after surgery. Data is presented as percentage. The prevalence of CPSP in group TP showed no significant difference compared with group P at 3 months and 6 months after surgery (all *P* > 0.05). CPSP = chronic postsurgical pain. Group P=PCIA only, Group TP = PCIA and TAP block

The prevalence of CPSP at 3 months and 6 months after surgery were summarized in Fig. 3. Among 307 patients, CPSP occurred in 62 patients (20.2%) at 3 months after surgery and 42 patients (13.7%) at 6 months after surgery in our patients. The prevalence of CPSP was 19.5% (25/128) in group TP and 20.7%(37/179) in group P at 3 months after surgery; 13.2% (17/128) patients suffered from CPSP in group TP and 13.9% (25/179) at 6 months after surgery. Both at 3 months and 6 months after surgery, there was no statistical difference of the prevalence of CPSP between the two groups (3 months, *P* = 0.806, 6 months, *P* = 0.863, respectively) .

## Discussion

Our research assumptions were partially supported. TAP block not only significantly decreased the intraoperative opioids, but also lessen pain intensity at 24 h and 3 months after colorectal surgery; however, such an effect did not occur at other time point.

In group TP, patients received bilateral TAP block before surgery, the NRS score both at rest and during movement at 24 h after surgery was significantly lower than that in group P. This result was similar to some studies, which showed that TAP block with levobupivacaine could reduce postoperative visual analogue pain scores in the first 24 h after major abdominal surgery, [17] and TAP block provided superior analgesia with lower pain scores at rest and movement in the first 24 h after colorectal surgery, when compared to PCA alone [18]. In our study, TAP block significantly decreased intraoperative sufentanil use, but there was no significant difference of sufentanil consumption at 24 h and 48 h after surgery and NRS score at 48 h after surgery between the two groups. Bhattacharjee et al. [19] also found that preincisional TAP block decreased intraoperative fentanyl requirements, and median time to request first postoperative analgesic was 290 min. According to McDonnell et al. [10] and our experience, the duration of TAP was approximately 8-16 h, with complete resolution at 24 h. Maybe this was the cause of the same opioid consumptions at 24 h and 48 h after surgery between the two groups. All suggested that TAP block could improve acute postoperative pain and the effect lasted at most 24 h.

In our study, the overall incidence of chronic pain was 20.2% at postoperative 3 months and 13.7% at postoperative 6 months. A prospective research documented that the prevalence of CPSP was 19% at 6 months after colorectal surgery [3]. A retrospective analysis confirmed that the prevalence of CPSP in patients with colorectal cancer was 17% at 38 months after laparoscopic colorectal surgery, [4] which was a little higher than ours at postoperative 6 months. These maybe have something to do with the difference of race and analgesia method.

However, there was no literature on how TAP block affects the incidence of CPSP in colorectal surgery. In our study, at 3 months after surgery, the prevalence of CPSP was 19.5% in group TP and 20.7% in group P; and at 6 months after surgery, the prevalence of CPSP was 13.2% in group TP and 13.9% in group P. There was no significant difference about the prevalence of CPSP between the two groups after elective colorectal surgery. Similarly, a prospective cohort follow-up study indicated TAP block did not influence the prevalence of CPSP at 6 months and 12 months after breast reconstruction [20]. A randomized controlled trial also demonstrated that TAP block did not decrease the incidence of CPSP at 6 months after cesarean delivery [21]. These told us that TAP block could not decrease the incidence of CPSP.

Although TAP block did not improve the incidence of chronic pain after colorectal surgery, pain scores at 3 months was lower in group TP than that in group P, which means TAP block has positive effect on CPSP. A randomized controlled trial [14] showed that TAP block could not reduce the incidence of chronic pain after inguinal hernia repair similar to ours but they differed in reduction of the pain scores in rest and movement at 6 months.

Our study has some limitations. The design of the retrospective study made it difficult for us to strictly exclude the use of postoperative non-opioid analgesics, which may also influence postoperative pain scores. In addition, most patients in this study had been operated by the laparoscopic approach and the incidence of chronic pain was not high after intervention, which might be the cause of negative long-term results.

## Conclusions

In conclusion, TAP block could relieve pain movement score at 3 months after surgery, but could not reduce the incidence of CPSP at 3 months and 6 months after selective colorectal surgery. Prospective study is required to assess the effect of TAP block on CPSP for prolonged period.

## Data Availability

The analyzed data sets generated during the study are available from the corresponding author on reasonable request.

## Abbreviations

TAP: Transversus abdominal plane
CPSP: Chronic postsurgical pain
PCIA: Patient controlled intravenous analgesia
NRS: Numerical rating scale
